# To Save the Drowning

**Published:** 1875-09

**Authors:** 


### TO SAVE THE DROWNING.

We call attention to the following brief and intelligible directions for saving the lives of persons rescued from the water after they have become insensible:

1. Lose no time. Carry out these directions on the spot.

2. Remove the froth and mucus from the mouth and nostrils.

3. Hold the body, for a few seconds only, with the head handing down, so that the water may run out of the lungs and windpipe.

4. Loosen all tight articles of clothing about the neck and chest.

5. See that the tongue is pulled forward if it falls back into the throat. By taking hold of it with a handkerchfef it will not slip.

6. If the breathing has ceased, or nearly so, it must be stimulated by pressure of the chest with the hands, in imitation of the natural breathing; forcibly expelling the air from the lungs, and allowing it to re-enter and expand them by the elasticity of the ribs. Remember that this is the most important step of all.

To do it readily, lay the person on his back, with a cushion, pillow, or some firm substance under his shoulders; then press with the flat of the hands over the lower part of the breast bone and the upper part of the abdomen, keeping up a regular repetition and relaxation of pressure twenty or thirty times a minute. A pressure of thirty pounds may be applied with safety to a grown person.

7. Rub the limbs with the hands or with dry cloths constantly, to aid the circulation and keep the body warm.

8. As soon as the person can swallow, give tablespoonful doses of hot water, or hot coffee or tea.

9. Work deliberately. Do not give up too quickly. Success has rewarded the efforts of hours.

